# Human monkeypox: A review of the literature

**DOI:** 10.1371/journal.ppat.1010768

**Published:** 2022-09-22

**Authors:** Rozana El Eid, Fatima Allaw, Sara F. Haddad, Souha S. Kanj

**Affiliations:** Division of Infectious Diseases, Internal Medicine Department, American University of Beirut Medical Center, Beirut, Lebanon; University of Arizona, UNITED STATES

## Abstract

Monkeypox (MPX) has recently made international headlines for the rapid and simultaneous progression of the disease across the world. This review aims at summarizing the literature available as well as describing the evolution of the disease as it pertains to the cases today along with potential treatments and infection control strategies. To date, more than 76 countries have reported cases in more than 12,261 people. Before this, MPX was a rare zoonotic disease confined to endemic areas in Western and Central Africa with sporadic outbreaks namely in the United States, associated with the import of wild animals from Ghana. However, during the current outbreak, human-to-human transmission has become the primary mode of transmission, raising concerns for unaccounted community spread. Most of these patients did not travel to the endemic areas of Africa, suggesting possible previously underdetected community transmission. Observations from emergent cases have reported that the manifestations of the disease were sometimes atypical from what has been previously described. Young men who have sex with men seem to be the population most vulnerable to infection. Though the disease is currently perceived to be mild in its clinical course, questions that remain unclear and warrant further investigation include potential of humans harboring a genital reservoir of the virus and the possibility of airborne transmission, which has implications for infection control and health of the community at large.

## Author summary

Except for a few limited incidents, monkeypox (MPX) transmission has been confined to endemic areas in Central and West Africa. However, since May 2022, multiple cases of MPX have been reported simultaneously across different regions of the world, stirring fears of a new pandemic. In this current outbreak, the clinical picture might be atypical and the disease should be suspected if epidemiological risk factors apply with different clinical presentations. Polymerase chain reaction (PCR) is used to confirm the diagnosis given its accuracy and sensitivity. Person-to-person transmission through direct contact with lesions and respiratory droplets is of particular concern, but there is an unclear consensus on the possibility of airborne transmission. The most affected group of individuals during this outbreak has been observed to be young men. There are no randomized control trials of MPX therapeutics in humans. Our review represents an important summary of the available evidence on MPX, focusing on its changing evolution in humans with the emergent cases, and describing its clinical characteristics, along with the risk factors for acquiring the disease, required infection control and prevention measures when dealing with patients, and the possible treatment options.

## Introduction

Monkeypox (MPX) has recently made international headlines and stirred public fears around a possible new global pandemic. To date, it has been reported across continents in more than 76 countries (Global.health Monkeypox (accessed on 2022 July 18)) and the Centers for Disease Control and Prevention (CDC) ramped up its alert to a level two [1]. Before this, monkeypox virus (MPXV) was a rare zoonotic disease confined to endemic areas in Western and Central Africa, which was described in humans for the first time in 1970 in a 9-month-old boy in the Democratic Republic of the Congo (DRC) [2]. Outside of endemic regions, the first reported MPX cases were in 2003 in the United States of America (USA) [3–5]. This outbreak was linked to the importation of Gambian giant rats, squirrels, and dormice [3–5]. Person-to-person transmission of MPX has only been recognized as a significant threat to global health when the 2018 outbreak in Nigeria exported the disease to sporadic areas globally, all of which were in travelers returning from Nigeria or their contacts [1,6–9].

In view of the recent increase in cases, we present a narrative review summarizing the previously available knowledge about MPX disease, focusing on its changing evolution in humans with the emergent new cases, and describing its clinical characteristics, along with the risk factors for acquiring the disease, required infection control and prevention measures when dealing with patients, and the possible treatment options.

## Virus and clades

MPX is caused by the MPXV, which belongs to the Poxviridae family and *Orthopoxvirus* genus. Additional members of this genus include vaccinia virus, variola (smallpox) virus, cowpox virus, and ectromelia (mousepox) virus. It is a large, enveloped, and brick-shaped double-stranded DNA virus. Like other poxviruses, it replicates within the cytoplasm of infected cells rather than in the nucleus [10].

Unlike smallpox, which was only reported as a human pathogen, MPXV has a range of hosts including animals and humans [11]. The virus falls into 2 distinct clades based on genetic and geographic variation: the Central African (or Congo Basin) clade and the West African clade [12]. The former clade appears to be more virulent based on higher observed mortality rates [13]. Although the Central African clade is more common, both outbreaks in the USA [3] and Nigeria [14] make up the largest part of the West African clade cases. The latter was also found in the travel-related cases in Israel, Singapore, and the United Kingdom [6,8,15]. With regard to the current outbreak, all cases reported in the UK have been confirmed as caused by the MPXV West African clade [16], and in fact all publicly available MPXV genomes from the 2022 MPX outbreak up to June have belonged to this clade [17]. However, viral genomic sequencing comparing the US MPXV sequences from May 2022 revealed 2 distinct lineages [17]. Additional phylogenomic analysis revealed that although 2022 MPXV (lineage B.1) clustered with 2018 to 2019 cases linked to an endemic country, it separates in a divergent phylogenetic branch, with the authors concluding that this likely indicates continuous accelerated evolution [18]. Historically, there is a possibility that human-to-human transmission that was reported in Africa at least 10 years ago went unrecognized with the virus spreading to various countries across the globe. However, this remains to be confirmed.

## Epidemiology and rising prevalence

There has been a greater than 10-fold increase in confirmed, probable, and/or possible MPX cases over the past 5 decades, from 48 cases in the 1970s to 520 cases in the 1990s, with the DRC being the country that is most affected [19]. In the first decade of the 2000s, MPX cases were only described in 3 countries in Africa but between 2010 and 2019, cases were reported in 7 African countries, which were Cameroon, Central African Republic (CAR), DRC, Liberia, Nigeria, Sierra Leone, and Republic of the Congo [19]. The collected data suggest that this trend represents actual disease increase and not merely a result of improved surveillance [20].

MPX was not reported outside Africa until the outbreak in the USA in 2003 when infected rodents were accidentally imported to the USA [5]. Back then, 71 cases of human MPX were identified in 6 states, with 34 laboratory-confirmed cases [3]. During that outbreak, the disease appeared to have a very low rate of person-to-person transmission [21]. Since then, transportation, sale, and release into the wild of prairie dogs and animals from Africa was prohibited by the CDC and Food and Drug Administration (FDA) [22]. Therefore, with the geographical spread and further resurgence in areas that had not reported cases of MPX in decades, concern for the public health impact of MPX has been growing even before the present-day outbreaks [19].

**Fig 1:**
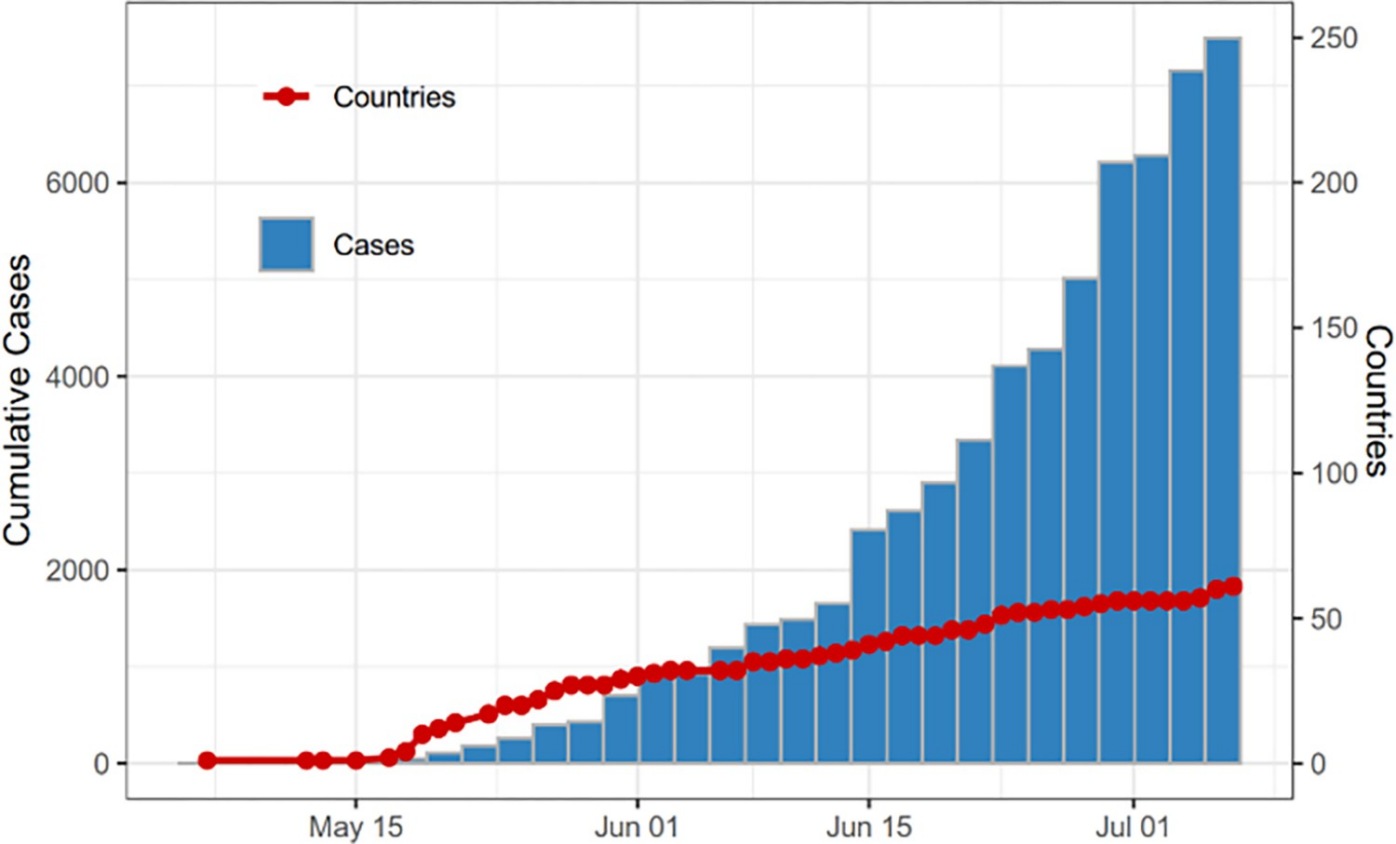
Number of cumulative confirmed cases and number of countries who have reported confirmed cases. Source: Global.health Monkeypox (accessed on 2022 June 9).

In the UK, from 2018 to 2021, 4 patients were diagnosed with travel-associated MPX with onward transmission to 3 patients [23]. This marked the first reported household cluster outside Africa. On May 7, 2022, the UK reported another new case of MPX outside Africa in a traveler returning from Nigeria. A few days later, 2 further cases who are part of the same family, which had not traveled and were not linked to the index case from Nigeria, were reported [16]. Since then, multiple cases have been identified, many with no known epidemiological links to the imported case from Nigeria or to the family cluster. What has been observed, however, is that these emerging cases were frequently men who have sex with men (MSM) that presented with symptoms such as a vesicular rash-like illness, lymphadenopathy, and fever [24,25]. Until June 6, more than 300 cases of MPX have been reported in the UK, and more than 30 cases have been reported in the US [1,26]. To date, more than 76 countries not usually endemic to MPXV and in some cases with no established travel link reported cases of the disease, bringing the total to more than 12,261 individuals affected [27,28]. Fig 1 shows the number of cumulative confirmed cases and number of countries who have reported confirmed cases (Global.health Monkeypox (accessed on 2022 July 18)).

While the previous outbreaks were able to be contained, the current cases showed a rapid human-to-human transmission, raising concerns for rapid community spread. Most of the patients did not travel to the endemic areas of Africa, suggesting possible previously underdetected community transmission. In addition, the fact that it occurred in multiple countries during the same period of time suggests multiple sources of introduction and transmission. More thorough investigations are needed to answer these questions associated with this outbreak.

## Reservoirs and transmission

Despite its name, monkeys are not the reservoir of MPXV. In fact, monkeys and humans are incidental hosts. The reservoirs are believed to be mainly rodents including squirrels and Gambian rats [29].

Animal-to-human transmission of MPX is well documented and usually happens through contact with an infected animal bodily fluid or through a bite [30]. In a study done based on the 2003 US outbreak, it was suggested that exposure be classified as “noninvasive” (touching an infected animal) or “complex” (invasive bite from an ill animal). Patients with complex exposures were more likely to develop systemic illness compared to those with noninvasive exposure [30]. The current outbreak has also raised concerns that MPXV could undergo “reverse zoonosis” by infecting wildlife outside of Africa, forming a reservoir that could be a seed for human outbreaks [31]. However, as of May 24, the European Food Safety Authority have stated that no pets or wild animals had been infected and none have been identified outside of Africa [32]. Furthermore, surveys of wild animals in Wisconsin and Illinois after the 2003 US outbreak never found any evidence of MPXV and none of the infected humans passed on the disease to other people [31]. On the other hand, 300 of the infected animals from Ghana and the exposed prairie dogs were never found [31]. Additionally, researchers have been able to intentionally infect many lab animals, including rabbits, hamsters, guinea pigs, animals that are common household pets [31].

Human-to-human transmission can usually occur through large respiratory droplets, with prolonged face-to-face contact, close contact with infectious skin lesions, or bodily fluids. Contaminated fomites objects, surfaces, such as living in the same household, sleeping on the same bedding, or eating/drinking from the same dishes of an infected individual are also considered risk factors for viral transmission [33]. The virus can also cross the placenta from the mother to the fetus [34]. It has been reported in a significant number of cases including nosocomial and household transmission [14,35,36]. Although there are questions whether MPXV can be transmitted via the airborne route, there currently exists no evidence to support this. In addition, the fact that many initial clinical presentations lack a prodromal phase and are confined to skin lesions suggests possible limited respiratory transmission and more skin-to-skin contact mode of transmission.

Though the cases of MPX associated with sexual intercourse are more likely to be the result of direct contact with skin lesions rather than being sexually transmitted, this latter theory has been postulated after the seminal fluid sampled from some cases was found to be positive for MPXV, with quantification cycle (Cq) values comparable to those obtained for nasopharyngeal swabs [37]. However, the clinical significance of this remains to be established.

## Demographic characteristics and risk factors

A recent systematic review found the weighted average of the median age of MPX infection in Africa has changed from 4 and 5 years in the 1970s and 1980s, respectively, to 21 years old between 2010 and 2019 [19]. Males are disproportionately affected [19]. The 22 studies included in this review reported a heterogeneous number of confirmed cases ranging from 1 to 785. No quality assessment could be performed. The preponderance of MPX cases (around 80% to 96%) have occurred in unvaccinated individuals for smallpox [19], with the highest percentage of vaccinated cases (21%) documented in the US outbreak [3].

During this year’s outbreak, a similar demographic pattern has been observed, where 99% of the reported cases were men with a median age of 37 years and interquartile range of 32 to 43 years [38]. The preponderance of MPX in this age group might be related to their smallpox vaccine naïve status.

Beer and colleagues [13] found that historically, the majority of outbreaks of MPX have occurred in rural populations living in small villages (less than 1,000 people) abutting or contained within humid evergreen tropical forests, named the “human–animal interface”. This could explain the observation of the preponderance of cases among young males, as they have been noted to trap and play with small rodents and their carcasses [37]. Indeed, an investigation into the outbreak in Nigeria from September 2017 through April 2018, which was the largest documented outbreak of MPX in West Africa, demonstrated that the initial cluster of 4 male individuals became ill after killing and eating a captured monkey from the area, which young boys regularly played with [37,39].

However, given that the current outbreak does not resemble previously observed outbreaks, the fact that males continue to be disproportionately affected warrants further explanation. Bragazzi and colleagues recently published a pooled analysis of cases up till 7 June 2022; the study revealed that all cases included in the analysis were males that have had unprotected sex with men [25]. This pattern was similarly observed in an analysis of cases from the UK, whereby sexual health histories identified links to various social encounters involving sexual intercourse, although no single factor or exposure that links the cases has been established [40,41]. Since unprotected sexual intercourse and sexual promiscuity are behaviors in which any person regardless of gender or sexual orientation can engage in, it remains unclear why MSM are at higher risk. One possibility could simply be the frequency with which this population group engages in these behaviors. Another possibility could be that the virus may be transmitted through sexual intercourse, combined with the observation that unprotected anal intercourse presents a higher likelihood of sexually transmitted infections than does unprotected vaginal intercourse, and therefore could lead to faster transmission among MSM [42].

## Clinical characteristics and diagnosis

It is important to note that for the cases prior to 2022 outbreak, the key characteristics are similar to the clinical course of ordinary discrete smallpox except for lymphadenopathy that distinguishes MPXV infection from smallpox [43]. In fact, the incubation period ranges from 5 to 21 days before the development of flu-like prodromal symptoms (e.g., fever, malaise, chills, headache, weakness, lymphadenopathy) [1]. A person with a complex exposure may have a shorter incubation period than a person with noninvasive exposure [30]. Following viral entry from any route, the virus replicates at the inoculation site and spreads to the local lymph nodes leading to an initial viremia phase that seeds the virus to other organs [44]. Over a period of 2 to 3 weeks, lesions typically develop starting from the oropharynx (enanthems), then a macular rash appears on the skin, starting on the face and spreading to the arms and legs, and then to the hands and the feet including palms and soles [44]. The rash then progress through 4 stages over a period of 2 weeks—macular, papular, vesicular, and pustular—before scabbing over and resolving [45]. Infected patients may be contagious from the prodromal phase until the last pustule scabs and falls off [1].

However, during the current outbreak, MPX appears to have a different clinical presentation compared to the previous outbreaks. First, many patients presented initially with rash without reporting having a prodromal phase of lethargy and asthenia, followed by the fever [41,46]. Some initial symptoms were confined to the pelvic region including proctitis, instead of starting with a rash on the face and before spreading to the extremities [22,34,47]. Several patients who presented with genital lesions had superimposed sexual transmitted infections [41,48]. One must maintain a high clinical suspicion after establishing an epidemiological link, while considering other sexual transmitted diseases, as those patients are expected to be seen in regular clinics. Moreover, and because of the variety in the initial rash presentation, a recent call was made for dental surgeons to be vigilant about MPX diagnosis since rash can also occur in the oral cavity [49]. A recent systematic review and meta-analysis about the clinical spectrum of the current reported MPX cases found that the distribution of the rash in the pelvic area and groins was significantly higher in European studies compared to the African studies, while lymphadenopathy and hospitalization was higher in the African studies [50]. However, fever, rash with centrifugal spread, pruritis, and lymphadenopathy remain critical clinical findings for MPX diagnosis [50]. More studies are needed to define the clinical evolution of the disease as most of the data are derived from case reports/case series and mostly from European countries.

The clinical picture is helpful in making the diagnosis. PCR is used to confirm the diagnosis given its accuracy and sensitivity [51–53]. Optimally, as recommended by CDC, samples should be taken from vesicles, pustules, or dry crusts. PCR from blood samples is less sensitive given the short duration of viremia compared to the timing of specimen collection [54]. However, MPXV PCR was detected in the semen and rectal mucosa of 3 patients from Italy [48]. In view of the evolution and the variety in the clinical spectrum of the disease, more studies are needed to evaluate the yield of PCR from non-skin sites as the clinical significance in terms of reservoirs and transmission is still to be determined.

IgM and IgG ELISA can be used as detection methods, particularly during the early phase of the infection [55]. The World Health Organization (WHO) recommends that these results should be interpreted carefully with the provided patient’s information that include date of fever onset, rash onset, specimen collection, current status of the rash, and age [28]. Point-of-care antigen detection for MPXV has been developed and may be used for rapid screening of patients [56]. However, as orthopoxviruses are serologically cross-reactive, antigen and antibody detection methods are not specific to MPX and are therefore not recommended for diagnosis or case investigation in resource-limited settings. [55,57]. Viral culture is not recommended as a routine diagnostic procedure [58].

## Case definitions

Case definitions are used for surveillance purposes to rapidly identify clusters and routes of transmission but should not be used to guide clinical management. These definitions are not standardized across sources and can be updated as more specific information becomes available.

## Treatment and prevention

There is no treatment currently available for human MPX and management remains supportive. Brincidofovir—a prodrug of cidofovir—and tecovirimat are 2 orally bioavailable drugs approved in the USA for the treatment of smallpox in case of a bioterrorism event [59–61]. These drugs have demonstrated efficacy against orthopoxviruses (including MPX) in animal models but were not assessed in human trials. Documented use of tecovirimat in the literature for complicated vaccinia and cowpox has been reported, with successful resolution and no concerning side effects [62,63]. A case series of 7 patients from the UK diagnosed with MPX between 2018 and 2021 reported that treatment with either brincidofovir or tecovirimat resulted in transient reductions in MPX viral PCR cycle thresholds, but these improvements were not durable or consistent between patients [23]. The significance of PCR positivity and threshold in MPX is still unclear, however. Randomized controlled trials are needed to determine whether the effects seen were attributed to treatment with these medications. Despite the potential use for antiviral drugs, symptomatic and supportive treatment remain the basis of management of MPX infection. Antiviral therapy may be indicated for patients with severe disease, immunocompromised, or in whom the infection is in atypical sites (eyes, mouth) or in the genital area [40].

There is evidence that smallpox vaccination with vaccinia virus is protective against MPX disease [64,65]. This was demonstrated in a study where human-to-human transmission was 5-fold less in vaccinated individuals (7.5%) compared to unvaccinated individuals (1.3%) [66]. However, since the eradication of smallpox in 1980, routine vaccination against smallpox was no longer indicated for the past 4 decades [19]. Thus, this cross-protective immunity from smallpox will be limited to older persons, and the worldwide population less than 40 years of age will no longer benefit from this protective immunity. During this current outbreak, WHO has issued that newer-generation smallpox vaccine (second or third) can be used as preexposure prophylaxis for healthcare workers at risks, and postexposure prophylaxis ideally within 4 days of first exposure [67]. Little is known about the efficacy of the vaccine and more clinical evidence is needed to provide strong recommendations [68]. Two vaccines are currently licensed by the US FDA for smallpox: ACAM2000 (IMVAMUNE) and JYNNEOS (IMVANEX). The effectiveness of these vaccines against other orthopoxviruses such as MPX could be inferred from observational studies; however, only JYNNEOS demonstrated efficacy against MPX in a clinical study [69]. At present, the Advisory Committee on Immunization Practices (ACIP) recommends that people who have a high risk of exposure to MPX get vaccinated with either ACAM2000 or JYNNEOS as preexposure prophylaxis [69].

In addition, the role of infection control strategies cannot be overstated. Rapid identification and appropriate isolation of patients, use of personal protective equipment by healthcare workers, hand hygiene and thorough contact tracing, including monitoring for secondary cases during the entire incubation period are the cornerstones of limiting the spread of disease [16]. During the current outbreak, one hospital using these interventions alone was able to protect medical staff and the broader community from further disease transmission [70]. Poxviruses are particularly resistant to drying and have increased temperature and pH tolerance enabling them to develop prolonged environmental persistence [16,71]. Cleaning of the room where a MPX case was present should be done without stirring a lot of dust or causing the formation of aerosols and should use regular cleaning products followed by disinfection using a 0.1% sodium hypochlorite (NaClO). Contaminated clothing and linens should be collected and washed at 60°C cycles [72].

There is still no strong evidence to suggest airborne transmission of MPXV, and this creates an unclarity in terms of infection control measures and implications for isolation and contact tracing. Thus, a recent guidance from the CDC recommends that contacts (including healthcare personals) should be instructed to monitor for symptoms for 21 days after their last exposure but should continue to carry their routine daily activities without isolation while refraining from donating blood, cells, tissue, and semen and refraining from breast feeding or engaging in sexual activities while under surveillance [73]. This indeed poses a challenge in healthcare facilities until more evidence is gathered about the virus mode of transmission, but for the time being, avoiding close contact of the exposed people with humans and pets is essential. Public health authorities have a major role in promptly identifying infected patients, isolating, treating, and administering vaccine when recommended. Innovative ways on monitoring the close contact to limit further spread of the virus are highly desirable. As with COVID-19, early actions allow a rapid response to the outbreak and have a potential great impact on containing the virus [74]. The microbiology laboratories have a great role in containing the outbreaks where there has been an increasing demand for their services, requiring accurate and rapid diagnostic testing tools [75].

## Conclusions

MPX is currently gaining attention worldwide, and people are concerned if these outbreaks can become a pandemic. For now, key gap information needs to be further studied to provide better answers such as the exact mode of transmission and the role of animal reservoirs. Sexual modes of transmission, genetic mutations, waning immunity from smallpox, and previous undetected cases of MPX warrant further investigation. Doctors should also keep in mind the atypical presentations and rely on the WHO and CDC criteria to guide patients and help in containing outbreaks.
