# Correction: Human monkeypox: A review of the literature

**DOI:** 10.1371/journal.ppat.1011008

**Published:** 2022-12-02

**Authors:** Rozana El Eid, Fatima Allaw, Sara F. Haddad, Souha S. Kanj

In the Treatment and Prevention section, there are errors in the seventh and eighth sentences of the second paragraph. The correct sentences are: Two vaccines are currently licensed by the US FDA for prevention of smallpox: ACAM2000 and JYNNEOS. The effectiveness of JYNNEOS against monkeypox was concluded from a clinical study on the immunogenicity of JYNNEOS and efficacy data from animal studies [69].
